# Remote Perimetry in a Virtual Reality Metaverse Environment for Out-of-Hospital Functional Eye Screening Compared Against the Gold Standard Humphrey Visual Fields Perimeter: Proof-of-Concept Pilot Study

**DOI:** 10.2196/45044

**Published:** 2023-10-19

**Authors:** Kang-An Wong, Bryan Chin Hou Ang, Dinesh Visva Gunasekeran, Rahat Husain, Joewee Boon, Krishna Vikneson, Zyna Pei Qi Tan, Gavin Siew Wei Tan, Tien Yin Wong, Rupesh Agrawal

**Affiliations:** 1 National University of Singapore Singapore Singapore; 2 National Healthcare Group Eye Institute Tan Tock Seng Hospital Singapore Singapore; 3 Singapore Eye Research Institute Singapore Singapore; 4 Raffles Medical Group Singapore Singapore; 5 Eye-ACP Duke-NUS Medical School Singapore Singapore; 6 School of Medicine University of New South Wales Sydney Australia; 7 Singapore National Eye Center Singapore General Hospital Singapore Singapore; 8 Tsinghua Medicine Tsinghua University Beijing China; 9 Lee Kong Chian School of Medicine Nanyang Technological University Singapore Singapore

**Keywords:** eye, screening, glaucoma, virtual reality, metaverse, digital health, virtual reality, visual impairment, visually impaired, functional testing, ophthalmologic, ophthalmology, remote care, visual field, HVF, perimetry test

## Abstract

**Background:**

The growing global burden of visual impairment necessitates better population eye screening for early detection of eye diseases. However, accessibility to testing is often limited and centralized at in-hospital settings. Furthermore, many eye screening programs were disrupted by the COVID-19 pandemic, presenting an urgent need for out-of-hospital solutions.

**Objective:**

This study investigates the performance of a novel remote perimetry application designed in a virtual reality metaverse environment to enable functional testing in community-based and primary care settings.

**Methods:**

This was a prospective observational study investigating the performance of a novel remote perimetry solution in comparison with the gold standard Humphrey visual field (HVF) perimeter. Subjects received a comprehensive ophthalmologic assessment, HVF perimetry, and remote perimetry testing. The primary outcome measure was the agreement in the classification of overall perimetry result normality by the HVF (Swedish interactive threshold algorithm–fast) and testing with the novel algorithm. Secondary outcome measures included concordance of individual testing points and perimetry topographic maps.

**Results:**

We recruited 10 subjects with an average age of 59.6 (range 28-81) years. Of these, 7 (70%) were male and 3 (30%) were female. The agreement in the classification of overall perimetry results was high (9/10, 90%). The pointwise concordance in the automated classification of individual test points was 83.3% (8.2%; range 75%-100%). In addition, there was good perimetry topographic concordance with the HVF in all subjects.

**Conclusions:**

Remote perimetry in a metaverse environment had good concordance with gold standard perimetry using the HVF and could potentially avail functional eye screening in out-of-hospital settings.

## Introduction

Population eye screening programs are critical for early detection of eye diseases to enable timely interventions and address preventable blindness [1,2]. The COVID-19 outbreak has resulted in massive disruptions to health care services [3,4], including the postponement of health care procedures as well as reduced patient uptake of elective services due to fears of contracting COVID-19 infection [5]. Despite the many successes in the continuity of care enabled by technology during the COVID outbreak, its disruptive impact still remains, particularly for screening health services [3,6]. In the field of ophthalmology, there is now a significant burden of “catch-up” screening, critical for the early detection of blinding diseases before the onset of irreversible visual impairment (VI) [7]. Glaucoma is one such leading cause of irreversible VI that is often detected late due to a lack of disease awareness and the often asymptomatic nature of early disease [8,9].

Disruptions in patient care due to the pandemic necessitated the redesigning of clinical workflows to ensure continued clinical service delivery across many medical disciplines, including ophthalmology [3,10,11]. This often required the incorporation of artificial intelligence, telehealth, and other related technologies across a spectrum of anatomical and functional eye screening processes [12]. However, existing remote perimetry solutions have several limitations that may impede translation, such as the need for variable and costly specialized hardware, dependence on trained operators for testing, lack of robust comparative evidence, as well as technical limitations in detecting mild visual field deficits [13,14]. These limitations present challenges for the interpretation of results from current remote perimetry solutions and are likely to introduce significant barriers to adoption, particularly in community-based primary care settings [9].

In a recently published user acceptance test (UAT) we demonstrated patient acceptance of gamified software applied for ophthalmology-related health promotion and education in a mixed reality metaverse environment using augmented reality or virtual reality (VR) [15]. The software was designed for compatibility with commercially available VR headsets that are easily accessible to patients. We subsequently reconfigured the software for application in suprathreshold perimetry testing in a VR metaverse environment to meet test requirements such as fixed pupil-screen distance and control of background luminance. This software, Perispace (PS), was designed to meet the eye care needs of community-based primary care settings and general practitioner clinics, to perform remote perimetry tests and triage referrals of patients to tertiary ophthalmology eye screening services.

In this proof-of-concept study, we performed a head-to-head comparison between the PS suprathreshold perimetry solution and the gold-standard Humphrey visual field (HVF) perimetry test.

## Methods

### Study Design

This was a prospective observational proof-of-concept study conducted at the National Health Care Group Eye Institute (NHGEI), Tan Tock Seng Hospital (TTSH), Singapore. Patients were included in this study only if they had a recent reliable HVF with tests performed within the prior 6 months. Criteria for reliable HVF test results in the manufacturer’s manual include less than 20% fixation losses, less than 33% false-negative errors, and less than 33% false-positive errors. Patients who were pregnant, unable to provide informed consent, or who had unreliable HVF perimetry results were excluded.

All eligible and willing participants received a comprehensive ophthalmic examination and a review of their recent gold standard HVF examination. HVF was performed using the 24-2 Swedish interactive threshold algorithm (SITA)–fast program [16] and visual field defects were classified as mild, moderate, or severe using the modified Hodapp, Parish, and Anderson criteria [17]. Where patients performed HVF tests in both eyes, only the right eye was selected for analysis. PS perimetry tests were performed only after HVF tests.

PS is a novel remote solution that replicates the photopic perimetry test of the HVF perimeter in a VR metaverse environment configured with clinical grade parameters including a background luminance of 10 cd/m^2^, age-adjusted stimulus luminance, and a stimulus angular size of 0.43°, corresponding to the Goldmann size III stimulus. Tests are performed by presenting stimuli to one eye at a time at random, to prevent malingering, with the VR headset calibrated using the Datacolor photometer. It has a wide, 120° field of view and uses PS software developed using the Unity engine (2020.3) to present test stimuli to one eye at a time at random.

### Key Outcome Measures

The primary outcome measure was the agreement in overall classification of perimetry result (“normality” of result) between the HVF 24-2 (SITA-fast) and the Perispace v1.1 algorithm. Automated classification of perimetry results by the HVF has been extensively described and validated in earlier research [16]. Due to the pathophysiology of glaucoma, the disease has the propensity to affect arcuate fibers from the optic nerve head instead of the papillomacular (PM) bundle fibers or nasal radiating fibers [18]. This is the basis for glaucomatous visual field defects—contiguous defects within the same hemifield with respect to the horizontal midline. Referral criteria applied to label an “abnormal” result based on topography in the PS perimetry results were based on the definition used in the landmark Ocular Hypertension Treatment Study (OHTS) [19]. Therefore, all patients with >2 abnormal points detected in PS that were vertically or horizontally contiguous in a given hemifield, were labeled “abnormal” and referred for tertiary assessment.

Secondary outcome measures for this study included the pointwise concordance of individual perimetry test points, visual comparison of perimetry topography maps, as well as the time taken for tests per eye, comparing results between PS and HVF perimetry tests. Pointwise concordance was calculated based on the agreement in automated classification of individual test points for all corresponding locations of the 24-2 test distribution, on both PS and HVF perimetry tests. Descriptive statistics are described, along with paired-sample 2-tailed *t* test to compare means of continuous variables. Statistical analysis was performed using SPSS (version 22; IBM Corp). P<.05 was considered statistically significant.

### Ethical Considerations

The study was reviewed and approved by the Institutional Review Board of TTSH (reference nummber: IRB201800601), Singapore for de-identified data collection without financial compensation. Consecutive willing patients were recruited and informed consent was obtained.

## Results

In this study, 10 consecutive subjects were recruited and analyzed. The average age of subjects was 59.6 (20.5) years (range 28-81 years). Of these subjects, 7 (70%) were male and 3 (30%) were female. Four (40%) subjects had pupil dilatation performed after the completion of HVF tests and before the conduct of PS tests. Demographics and clinical information are depicted in Table 1. Based on gold standard HVF perimetry tests, 2 (20%) subjects were found to have no visual field defect, 4 (40%) subjects with mild visual field defect, 2 (20%) subjects with moderate visual field defect, and 2 (20%) subjects with severe visual field defect.

**Table 1:** Demographics and clinical information of study participants.

Variable/category			Values
**Gender, n (%)**			
	Male	7 (70)	
	Female	3 (30)	
Age, (years) mean (SD)			59.6 (20.5)
Age range (years)			28-81
**HVF^a^ result, n (%)**			
	None	2 (20)	
	Mild	4 (40)	
	Moderate	2 (20)	
	Severe	2 (20)	
**Pupil dilation at the time of HVF and PS^b^ test, n (%)**			
	None	6 (60)	
	Before both HVF and PS	0 (0)	
	After HVF, before PS	4 (40)	
**Lens status, N (%)**			
	Normal	4 (40)	
	Cataract	4 (40)	
	Intraocular lens	2 (20)	

^a^Humphrey visual field.

^b^Perispace.

**Figure 1:**
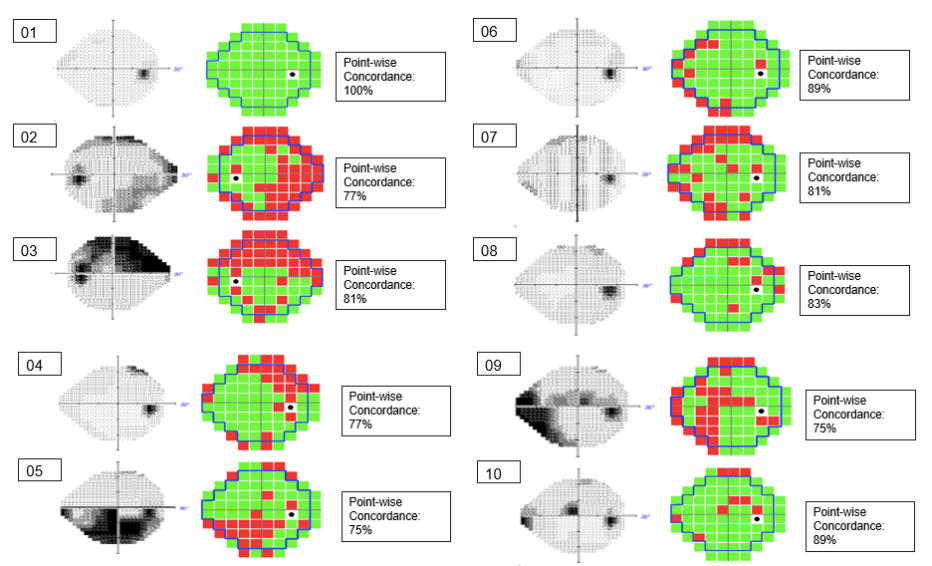
Point--wise concordance of Perispace v1.1 and Humphrey visual field (HVF) results with a 24-2 testing distribution. Corresponding perimetric topographic maps and point-wise concordance for automated classification of individual test points in all corresponding locations of the Swedish interactive threshold algorithm–fast testing distribution in the HVF results (left) and remote perimetry in a virtual reality–based metaverse environment using Perispace (right). If the concordance was good, then black areas of the HVF gray scale should appear as red areas of the Perispace topographic map. The letters A to J represent individual patients.

The agreement in overall classification of perimetry result normality between the HVF and PS was high (9/10, 90%). The pointwise concordance in the automated classification of individual test points was 83.3% (8.2%; range 75%-100%). The corresponding output perimetry greyscale topographic maps for HVF and that for PS had good topographic concordance for each of the 10 subjects, as depicted in Figure 1. The PS was reasonably able to delineate clinically significant visual field defects, such as superior and inferior arcuate scotomas (patients C and E), nasal step defects (patients B, I, and J), as well as central or paracentral defects (patients I and J), as illustrated in Figure 1.

The test time per eye was faster for PS than for HVF perimetry tests in 7 patients (7/10, 70%). The mean test time was 219.9 (29.4) seconds for the PS test, compared to 230.5 (29.8) seconds for the HVF. However, there was no statistically significant difference in mean test time (*P*=.25).

## Discussion

### Principal Findings

Clinical service disruptions arising as a result of the COVID-19 pandemic have exacerbated existing capacity shortages in community eye screening [20]. PS is a novel remote perimetry solution that replicates photopic perimetry calibrated with clinical grade configurations that address constraints with existing solutions. This study shows that remote perimetry using PS in a VR metaverse environment demonstrates good overall agreement, pointwise concordance, and topographic map concordance (Figure 1) when compared with the gold standard HVF 24-2 SITA-fast perimetry test.

These were critical outcome measures prioritized that were based on consensus from discussions with general practitioners and ophthalmic surgeons, to ensure out-of-hospital remote perimetry remains aligned with gold-standard perimetry and existing clinical referral workflows. Novel remote solutions that emerged in recent years have also demonstrated good performance when compared to the HVF [9]. Current leading solutions include the Eyecatcher in the United Kingdom, C3 Field Analyzer (CFA) in India, and VisuALL in the United States, among others [21-24]. Benefits of these perimetry solutions over the gold standard HVF include lower cost, portability, convenience, and patient acceptance [21,24,25]. Existing remote perimetry tools can be broadly classified into VR-based and non–VR-based solutions.

### Limitations

The VisuALL and CFA tests are VR-based perimetry solutions using suprathreshold and threshold test strategies, respectively. Both tests exhibited good agreement in overall classification of perimetry result normality and concordance of perimetry topographic maps when compared against the HVF [22-24]. However, both the VisuALL and CFA tests appear to face limitations with regard to background and stimulus luminance. Both tools use background luminance in the scoptopic range, due to the limitations in the stimulus range of their hardware headset. This includes a background luminance of 3 cd/m^2^ reported for the VisuALL device and 4 cd/m^2^ reported for the CFA device, in contrast with the 10 cd/m^2^ of the gold standard HVF, which lies in the photopic range, approximating testing for daylight vision. The use of scotopic background luminance obfuscates the comparison of results with the HVF test and increases the risk of error due to the increased influence of absolute stimulus intensity on perception, being unable to account for pupil size and optic media effects [26].

The VisuALL is a promising solution that can be performed on the Pico VR headset with excellent performance, demonstrated previously [22,23]. However, the Pico headset only has a documented field of view of 100°, in comparison with the 108° required for the bilateral 24-2 HVF test distribution [23]. Moreover, the adequacy of the stimulus range with the headset is unclear. It was reported as 120 cd/m^2^ in one trial [22], but a later study reported a maximum intensity of 110 cd/m^2^ when plotted against RGB values [23]. On the other hand, the CFA was occasionally unable to identify deficits that matched the HVF test potentially due to inaccurate stimulus luminance, given that investigators used the Luxmeter to perform calibration to 60 cd/m^2^ to approximate a fixed deficit of 18 dB, instead of using a photometer [24].

Non-VR headsets have other hardware limitations. The Eyecatcher perimetry solution uses a similar suprathreshold test strategy as the PS. However, it is unable to detect the 4 most central points of the 24-2 perimetry test distribution, due to the spatial imprecision of the sensors. This precludes testing in the crucial central-vision region, which can be impaired first in early glaucoma [21,27]. Of note, the Eyecatcher demonstrated good agreement with the HVF in overall classification of perimetry result normality, concordance of the individual test points with a mean of 83% (similar to the mean of 83.3% in PS), and concordance of perimetry topographic maps [27]. However, the pointwise concordance with the Eyecatcher ranged from 50%-97%, compared to that for PS which was 75%-100%. This pointwise variation is similar to the spectrum of test-retest variability of HVF perimetry that has been reported on repeating the test due to contributing factors such as loss in patient attention, with worsening variability at the midrange of vision sensitivity [28]. The authors of the Eyecatcher study also reported that hardware limitations such as limited spatiotemporal precision and imperfect gaze-calibration may contribute to the pointwise variation [27]. Future research is needed to compare test-retest variability between perimetry devices and develop solutions to reduce variation such as by improving engagement using gamification and immersion in metaverse environments to maximize patient attention and effort with each run of the perimetry test.

In addition to the above technical limitations, current solutions require skilled operators to reinforce testing instructions to ensure reliable results, as well as to operationalize requirements such as gaze calibration or eye patching for monocular testing. In addition, several non–VR-based solutions such as the Eyecatcher are tablet based and require additional specialized head- or eye-tracking hardware [29]. Moreover, unlike VR headsets, these do not have a closed cupola and have a restricted spatial range [21]. This necessitates correction for eye movements, resulting in test-retest variations in the shape and extent of the testing and output topographic map [27]. Therefore, they require scaling of testing stimuli to screen-size based on testing set-up (which may vary from one health care setting to the next), a dedicated room to prevent visual distractions, and adjustable lights dimmed for testing [21,27].

Therefore, existing remote perimetry solutions described in the literature may not be suitable for primary care settings that are often manpower-, resource-, and space-constrained [29]. The strengths of the PS solution are that it addresses some of these limitations through automation of operator requirements such as monocular patching through algorithmic randomized differential testing of the patient’s eyes, calibration of testing parameters with the gold standard HVF using a photometer, as well as a closed cupola to provide sufficient spatial range, adequate ambient light control, and prevent external visual distractions. Moreover, with the automation of perimetry testing in a metaverse environment, this work has paved the way to decentralize the availability of crucial functional vision testing into out-of-hospital settings to overcome the limitations of physical space and infectious disease transmission risk in hospital settings [20]. The limitations of this proof-of-concept study include a small cohort and a lack of control group for analysis. Despite these limitations, the results of this study justify further research using case-control methodology in larger, prospectively recruited cohorts stratified based on well patients or those with relevant comorbidities. These studies should be designed to examine the performance of remote perimetry solutions in discriminating patients requiring referral for further assessment or interventions, and standardized testing procedures (such as lack of pupil dilatation).

### Conclusions

In conclusion, remote perimetry using PS in a VR metaverse environment had good performance when compared against gold-standard HVF perimetry, based on overall agreement, pointwise concordance, and topographic map concordance. PS has several design benefits over existing remote perimetry solutions, that have been customized to avail perimetry for functional eye screening in out-of-hospital settings. This can help to facilitate the early detection of patients with VI in the community and prompt early referrals for formal evaluation in hospital settings to help address global trends in increasing preventable blindness [1].

## Abbreviations

CFA: C3 field analyzer
HVF: Humphrey visual field
NHGEI: National Health Care Group Eye Institute
NHI: Northeast Health International
OHTS: Ocular Hypertension Treatment Study
PM: papillomacular
PS: Perispace
SITA: Swedish interactive threshold algorithm
TTSH: Tan Tock Seng Hospital
VI: visual impairment
VR: virtual reality

## Data Availability

The data set for this study is not publicly available due to local privacy regulations and a lack of patient consent for data sharing with public repositories.
